# An improved modified early warning score that incorporates the abdomen score for identifying multiple traumatic injury severity

**DOI:** 10.7717/peerj.10242

**Published:** 2020-10-27

**Authors:** Xiaobin Jiang

**Affiliations:** Emergency Department 1, Shanghai Ninth People’s Hospital, Shanghai Jiaotong University School of Medicine, Shanghai, China

**Keywords:** Multiple trauma, Injury severity, Modified early warning score

## Abstract

**Background:**

Rapid identification of trauma severity is essential for the timely triage of multiple trauma patients. Tools such as the modified early warning score (MEWS) are used for determining injury severity. Although the conventional MEWS is a good predictor of mortality, its performance assessing injury severity is moderate. This study hypothesized that adding an injury site severity-related score (e.g., abdomen score) may enhance the capability of the MEWS for identifying severe trauma.

**Method:**

To validate the hypothesis, we propose an improved modified early warning score called MEWS-A, which incorporates an injury site-specific severity-related abdomen score to MEWS. The utility of MEWS and MEWS-A were retrospectively evaluated and compared for identifying trauma severity in adult multiple trauma patients admitted to the emergency department.

**Results:**

We included 1,230 eligible multiple trauma patients and divided them into minor and severe trauma groups based on the injury severity score. Results of logistic regression and receiver operating characteristic (ROC) curve analyses showed that the MEWS-A had a higher area under the ROC curve (AUC: 0.81 95% CI [0.78–0.83]) than did the MEWS (AUC: 0.77 95% CI [0.74–0.79]), indicating that the MEWS-A is superior to the MEWS in identifying severe trauma. The optimal MEWS-A cut-off score is 4, with a specificity of 0.93 and a sensitivity of 0.54. MEWS-A ≥ 4 can be used as a protocol for decision-making in the emergency department.

**Conclusions:**

Our study suggests that while the conventional MEWS is sufficient for predicting mortality risk, adding an injury site-specific score (e.g., abdomen score) can enhance its performance in determining injury severity in multiple trauma patients.

## Introduction

In China, multiple trauma is one of the leading causes of death (Wang, Pan & Pan, 2017; Yin, Liang & Liu, 2015). Trauma resulting from motor vehicle and work-related accidents is responsible for more than 400,000 deaths in China annually; of these cases, approximately 1.0–1.8% involve multiple trauma (Zhang, Hong & Gregory, 2017; Yingcheng et al., 2014). The management of multiple trauma presents important challenges to the emergency department (ED) in China owing to the high mortality risk associated with such injuries.

Rapid identification of trauma severity is essential for the timely triage of multiple trauma patients. However, the assessment of multiple trauma severity might require a sophisticated whole-body medical examination by a specialist, which is time-consuming. To overcome this challenge, scoring systems that can predict mortality risk and assess injury severity for trauma patients in the emergency department have been developed.

Trauma scoring systems can be broadly categorized into anatomical and physiological scoring systems. Anatomical trauma scores are mainly injury site-dependent scores such as the abbreviated injury scale (AIS) score (Loftis, Price & Gillich, 2018) and the AIS-derived injury severity score (ISS) (Baker et al., 1974), which evaluates the injury severity for each site and provides a weighted score for the sites with the most serious injuries. Although such tools are effective, they also require trauma specialists to examine each injury site, which is, again, time-consuming and impractical in an ED.

Physiological trauma scoring systems include the revised trauma score (RTS) (Alvarez et al., 2016), APACHE II (Godinjak et al., 2016), sepsis-related organ failure assessment (SOFA) score (Raith et al., 2017), and emergency trauma score (ETS) (Raum et al., 2009). These scores incorporate age, physiological variables (e.g., heart rate), and neurological variables (e.g., the Glasgow coma scale (GCS)) to identify injury severity. RTS may help in decision-making for hospital discharge and intensive care unit (ICU) admission (Mansour, Eisha & Asaad, 2019), but its accuracy in predicting mortality decreases with increasing age (Kojima et al., 2019) and exhibits poor correlation with anatomical injury severity (Galvagno Jr et al., 2019). APACHE II has proven to be a good predictor of mortality among brain injury patients (Nik et al., 2018), but it is inefficient for trauma triage in EDs because it has too many variables; its calculation requires 14 variables. The SOFA score has been used to quantify organ function and may predict mortality in ICU patients (Bingold et al., 2015). However, it is not well known among emergency doctors and its use is inconvenient because several days may be required for calculations aimed at verifying whether patients fulfill the strict criteria and require laboratory tests (Machado et al., 2016). The ETS is a simpler predictor of in-hospital mortality in trauma patients (Park et al., 2017), but a duration of 30 min may be required to measure the parameters (Raum et al., 2009). Overall, these scoring systems are reliable tools for identifying trauma severity, but they are also inconvenient to calculate and measure. The inherent complexity of sophisticated scoring tools makes their application as a rapid risk stratification tool that can be applied in a regular ED setting challenging.

Given the simplicity and speed required for tools to be effect in triaging multiple trauma patients in EDs, tools such as the modified early warning score (MEWS) (Bozkurt et al., 2015) have now been widely applied in EDs, particularly in China (Xie et al., 2018). The MEWS has been used for many purposes: to predict in-hospital mortality (Xie et al., 2018), determine hospital admission (Wei et al., 2019), identify critically ill patients (Kruisselbrink et al., 2016), and predict injury severity and need for ICU admission (Salottolo et al., 2017).

However, our previous study (Jiang, Jiang & Mao, 2019) suggests that although the MEWS is effective for predicting mortality, it is not an excellent predictor of trauma severity, as its area under the receiver operating characteristic (ROC) curve (AUROC) for trauma severity prediction is less than 0.8. Wong et al. (2016) reported that adding an evaluation variable related to the injury site severity to an injury severity scoring system may improve the performance of the scoring system; thus, we hypothesized that adding an abdomen score, a variable for evaluating the severity of injury to the thorax and abdomen, to the MEWS may enhance its ability to predict injury severity of multiple trauma patients.

To validate the hypothesis, we proposed a scoring system called the MEWS-A, which combines the conventional MEWS with an abdomen score. We retrospectively evaluated and compared the utility of the MEWS and MEWS-A for identifying trauma severity of multiple trauma patients admitted to the ED.

## Materials and Methods

### Ethical statement

The study protocol was approved by the Ethics Committee of Shanghai Ninth People’s Hospital (approval no.: 2018-146-T132 and 2020-2018-146-T132-1), which waived the requirement to obtain informed consent.

### Settings and subjects

The study cohort included adult multiple trauma patients who were consecutively admitted to the author’s institution between January 2014 and June 2018. The institution is an urban emergency medical center, admitting approximately 20,000 trauma patients each year, of which approximately 3,000 admissions present with multiple trauma. As in Frink et al. (2017), we defined multiple trauma as two or more body region injuries where one or a combination of multiple injuries may endanger the patient’s life. We determined multiple trauma by reviewing medical records and computed tomography (CT).

Among all adult multiple trauma patients, those who had a pre-injury medical history or dysfunction were excluded because it would be challenging to identify outcomes directly related to injury severity (Jiang, Jiang & Mao, 2019). The study cohort was divided into minor and severe trauma groups according to ISS scores (ISS score <16 and ≥16, respectively). ISS ≥16 indicates that patients have a greater need for trauma care (Galvagno Jr et al., 2015), and it is a conventional threshold for defining severe trauma (Kehoe et al., 2015).

### MEWS and MEWS-A

Table 1 shows the MEWS and MEWS-A scoring systems, which are used to assess the trauma severity of the included patients and compared. The conventional MEWS consists of four vital sign variables: systolic blood pressure (SBP), heart rate (HR), respiratory rate (RR), temperature (T), and one neurological variable, AVPU. AVPU measures the level of consciousness and has four possible outcomes: alert (A), verbal (V), pain (P), and unresponsive (U). As reported previously (Mitsunaga et al., 2019), the AVPU score is derived from the GCS as follows: *A* = 14–15, *V* = 9–13, *P* = 4–8, and *U* = 3. The MEWS is reported as an effective tool in identifying the risk of mortality (Kruisselbrink et al., 2016), but it is less sensitive in identifying trauma severity (Jiang, Jiang & Mao, 2019).

**Table 1:** MEWS and MEWS-A score.

	**Score**			
**Variable**	**0**	**1**	**2**	**3**
Systolic blood pressure (mmHg)	101–199	81–100	70–80	<70
			≥200	
Heart rate (beats/min)	51–100	40–50	<40	≥130
		101–110	111–129	
Respiratory rate (cycles/min)	9–14	15–20	<9	≥30
			21–29	
Temperature (°C)	35–38.4		<35	
			≥38.5	
AVPU score	Alert	Reacts to voice	Reacts to pain	Unresponsive
Abdomen score	Abdomen and	Abdomen or	Abdomen rigid or	
	Thorax non-tender	Thorax tender	Flail chest	

The MEWS-A is generated by adding an abdomen score variable to the MEWS in order to improve its ability to assess injury severity. Designing new criteria (e.g., score range and calculation method) for the abdomen score is challenging; hence, we used an abdomen score that has been proposed in another scoring system, the Circulation, Respiration, Abdomen, Motor, Speech (CRAMS) scale (Gormican, 1982), which has been proven valid and reliable for assessing mortality risks of multiple trauma patients (Chen et al., 2017). Further, the abdomen score is preferable in EDs because it is convenient to measure and easy to remember. Abdomen score can be assessed by checking for tenderness (e.g., pain or discomfort) in the abdomen and thorax by palpation with no medical devices. Specifically, as shown in Table 2, the abdomen score ranges from 0 to 2: 0, 1, and 2 points represent conditions of non-tender abdomen and thorax, tender abdomen or thorax, and rigid abdomen or flail chest, respectively.

**Table 2:** Characteristics of the study cohort.

	**Total**	**Minor trauma**	**Severe trauma**	***P*-value**
No.	1230	475	755	
Male, n (%)	904 (73.5)	323 (68.0)	581 (77.0)	0.001
Age (years)	48 (38, 59)	50 (39, 60)	48 (38, 58)	0.099
Temperature (°C)	37.0 (36.7, 37.0)	37.0 (36.8, 37.0)	36.8 (36.5, 37.0)	<0.001
Heart rate (beats/min)	85 (75, 96)	83 (76, 90)	85 (75, 100)	0.012
Respiratory rate (cycles/min)	20 (18, 21)	20 (19, 20)	20 (18, 21)	0.012
Systolic blood pressure (mmHg)	130 (115, 147)	136 (121, 150)	127 (107, 145)	<0.001
AVPU	0 (0, 1)	0 (0, 0)	1 (0, 2)	<0.001
LOS	12.0 (5.0, 21.0)	9.0 (6.0, 15.0)	15.0 (4.0, 26.0)	<0.001
Time of transport (hour)	2.0 (1.0, 3.0)	2.0 (1.0, 3.0)	2.0 (1.0, 3.0)	<0.001
Cause of injury, n (%)				<0.001
Motor vehicle crashes	683 (55.5)	217 (45.7)	466 (61.7)	
High fall	222 (18.0)	69 (14.5)	153 (20.3)	
Crushing injury	41 ( 3.3)	11 ( 2.3)	30 ( 4.0)	
Cut/piercing injury	45 ( 3.7)	29 ( 6.1)	16 ( 2.1)	
Burn	4 ( 0.3)	2 ( 0.4)	2 ( 0.3)	
Tumble injury	169 (13.7)	112 (23.6)	57 ( 7.5)	
Striking injury	66 ( 5.4)	35 ( 7.4)	31 ( 4.1)	
Primary injury site–n (%)				<0.001
Face	19 ( 1.5)	17 ( 3.6)	2 ( 0.3)	
Head and neck	537 (43.7)	102 (21.5)	435 (57.6)	
Thorax	318 (25.9)	129 (27.2)	189 (25.0)	
Abdomen and visceral pelvis	111 ( 9.0)	42 ( 8.8)	69 ( 9.1)	
Bony pelvis and extremities	243 (19.8)	184 (38.7)	59 ( 7.8)	
External structures	2 ( 0.2)	1 ( 0.2)	1 ( 0.1)	
Discharge status, n (%)				<0.001
Died in the hospital	185 (15.0)	2 ( 0.4)	183 (24.2)	
Discharged home	583 (47.4)	303 (63.8)	280 (37.1)	
Discharged against medical advice	73 ( 5.9)	29 ( 6.1)	44 ( 5.8)	
Discharged home with recommended	344 (28.0)	112 (23.6)	232 (30.7)	
self-care				
Transferred to another hospital	45 ( 3.7)	29 ( 6.1)	16 ( 2.1)	
Score				
MEWS	2 (1, 3)	1 (1, 2)	2 (1, 4)	<0.001
MEWS-A	3 (1, 4)	1 (1, 2)	4 (2, 5)	<0.001

### Statistical analysis

Statistical analysis was conducted on both scoring systems to evaluate their performance in assessing trauma severity in multiple trauma patients using R, version 3.5.2 (R Foundation for Statistical Computing, Vienna, Austria). Multiple imputation was applied for the included patients who had missing values for one to three vital signs. The MEWS and MEWS-A score, shown in Table 1, were then computed for the minor and severe trauma groups.

Numerical and categorical outcomes are presented as medians (interquartile ranges [IQRs]) and frequencies (percentages), respectively. Categorical outcomes were analyzed by Pearson’s chi-square test. Normality tests were performed on numerical outcomes. Normally distributed numerical outcomes were analyzed by the *t*-test while non-normally distributed outcomes were analyzed by the Wilcoxon-Mann–Whitney test.

To evaluate the scoring systems’ performance in identifying trauma severity, the AUROC for the following logistic regression model was compared for the two scoring systems.

(1) \begin{eqnarray*}y= \frac{1}{1+{e}^{-(b+wx)}} \end{eqnarray*}

where *b*, *w*, and *x* indicate the bias, the weight, and the score, respectively.

To find the optimal decision threshold for identifying severe trauma, optimal cut-off points of both scoring systems were calculated. Further, the corresponding accuracy rate, sensitivity, and specificity at the point was analyzed.

**Figure 1:**
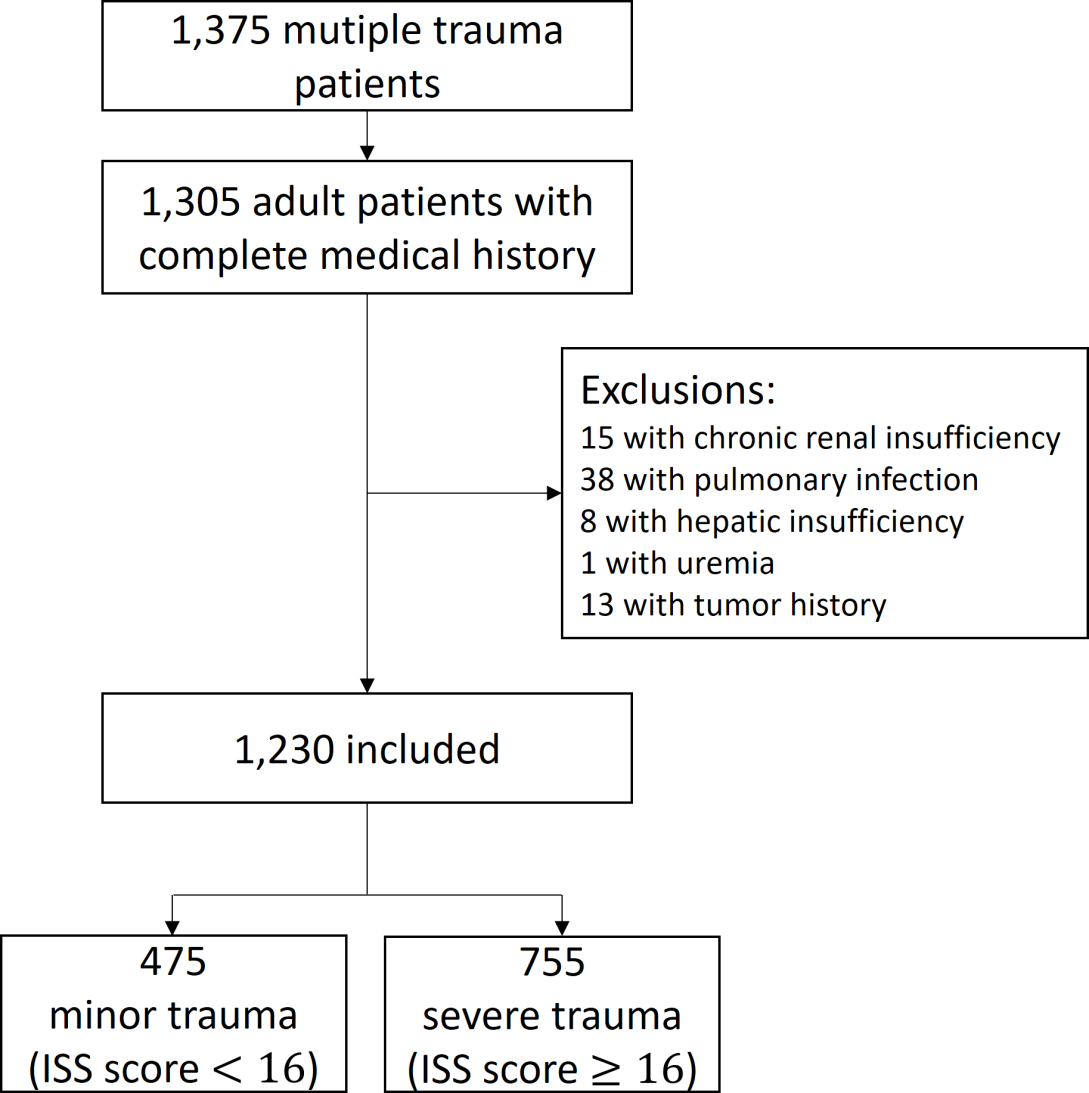
Study cohort.

## Results

### Setting and subjects

Figure 1 summarizes the study cohort. In total, 1,230 adult patients presenting with multiple trauma met the inclusion criteria. The minor injury and severe injury groups included 475 (38.6%) and 755 (61.4%) cases, respectively. Of 1,230 patients, 52 (4.2%) had missing vital signs data, among whom 30 had missing data of HR, RR, and SBP, 9 had missing data of SBP, 8 had missing data of RR and SBP, 2 had missing values of RR, 1 had missing data of HR and SBP, 1 had missing data of T and SBP, and 1 had missing data of T.

Normality testing results showed that distributions of all numerical variables could not be approximated as normal distributions. As shown in Table 2, male patients, whose median (IQR) age was 48 (38–59) years, made up 73.5% of the sample. Most patients (90.4%) were transported to the ED by an ambulance. Comparing the length of stay (LOS) of the two groups, as expected, severe trauma patients had longer stays (15 [4–26] vs. 9 [6–15] days) in the hospital. Patients’ injuries were primarily the result of motor vehicle accidents (55.5%). Most minor trauma patients suffered from injuries to the bony pelvis and extremities (38.7%) while most severe trauma patients suffered from injuries to the head and neck (57.6%).

The median MEWS and MEWS-A score (IQR) of the two groups were 1 (1-2) vs. 2 (1-4) and 1 (1-2) vs. 4 (2-5), respectively (*p* < 0.001).

### Performances of the MEWS and MEWS-A score

**Figure 2:**
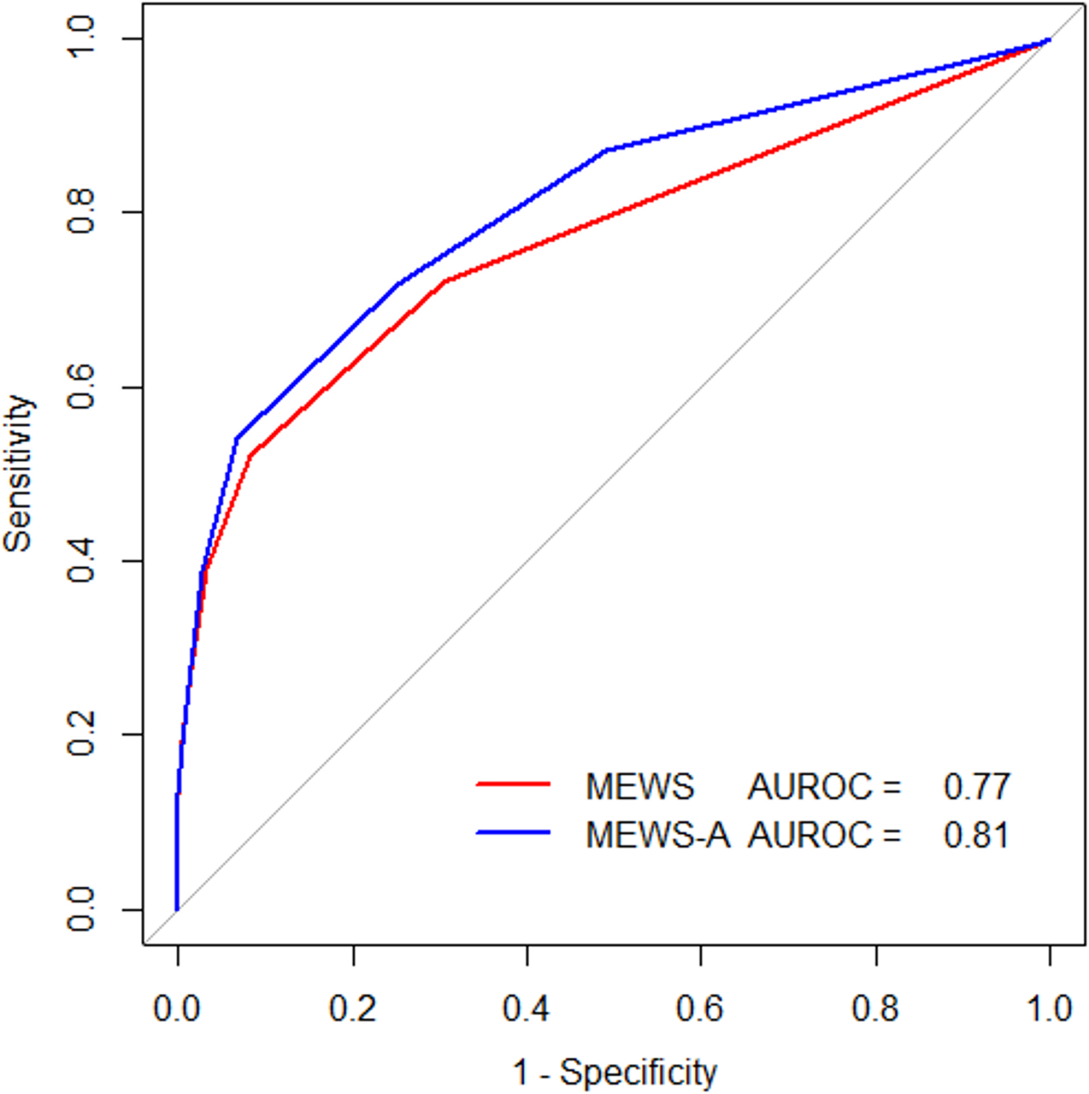
Receiver operating characteristic (ROC) curves for MEWS and MEWS-A.

As shown in Fig. 2, the MEWS-A has a greater AUROC value (AUROC = 0.81, 95% CI [0.78–0.83]) than the MEWS (AUROC = 0.77, 95% CI [0.74–0.79]) for multiple trauma severity assessment, showing that incorporating the abdomen variable improves the trauma severity assessment performance. DeLong’s test was used to compare the two ROCs and the *p*-value was *p* < 0.001.

The optimal cut-off values of ≥3 for the MEWS and ≥4 for the MEWS-A score were established. The corresponding accuracy, sensitivity, and specificity rates of the MEWS and MEWS-A score were 0.67 vs. 0.69, 0.52 vs. 0.54, and 0.92 vs. 0.93, respectively.

## Discussion

This study aimed to validate the hypothesis that adding an injury site severity score (abdomen score) to the conventional MEWS may improve its ability to identify trauma severity. Our results demonstrate that the MEWS-A score had a higher AUROC value (AUROC = 0.81) for trauma severity assessment than the conventional MEWS (AUROC = 0.77), indicating that incorporating the abdomen score improves the performance of the conventional MEWS.

The conventional MEWS has been applied for identifying mortality risk and determining trauma severity. The MEWS was previously reported to be an excellent predictor of in-hospital mortality because the MEWS achieves high area under ROC (AUROC ranges from 0.83 to 0.90 in Xie et al. (2018), Jiang, Jiang & Mao (2019), and Smith et al., 2013), although the performance may decrease for elderly patients (Mitsunaga et al., 2019). However, the performance of the MEWS in identifying injury severity is moderate (AUROC <0.8). Statistical analyses (Salottolo et al., 2017) showed that the MEWS is less associated with severe injury than in-hospital mortality. (Jiang, Jiang & Mao, 2019) also showed a lower AUROC value (AUROC = 0.77) of identifying trauma severity. Similarly, our results showed that the MEWS is a moderate predictor (AUROC = 0.77) for assessing injury severity.

By adding the abdomen score to MEWS, its ability to identify injury severity was improved. The conventional MEWS includes vital signs and AVPU. The vital signs may correlate well with mortality, but they show a poor correlation with injury severity. Even severe trauma patients may have a low MEWS and stable physiological measures (Gottschalk et al., 2006). As reported previously, no physiological measure has a sensitivity high enough for a negative result (e.g., normal physiological value) to be confidently used for concluding that a patient is not seriously injured (Annette et al., 2018). In addition, injury sites can be anatomically categorized into 6 body regions: head, face, chest, abdomen, extremities (including pelvis), and external (Quah, 2016). The AVPU score is derived from the GCS, a triage tool that assess traumatic brain injury (Gill et al., 2006). Hence, the AVPU score could play a major role in identifying trauma severity at the head and neck. With the additional incorporation of an abdomen score, the MEWS-A score may help in evaluating injury severity in the chest and abdomen, leading to better performance in identifying overall injury severity.

Although the MEWS-A has a higher AUROC value, the specificity (correctly identified as minor trauma) and sensitivity (correctly identified as severe trauma) are marginally higher than those of the conventional MEWS. Several further modifications may improve the function of MEWS-A and will be addressed in future research. First, replacing AVPU with GCS may improve the function because AVPU is estimated from GCS and the association of AVPU with GCS may vary with age (Nuttall, Paton & Kemp, 2018). Second, the proposed MEWS-A has the variables to assess trauma severity of the head, face, chest, and abdomen, but has no variables to evaluate severity of injuries to the extremities and external region. One solution to this is to add an AIS score for extremities and external region (Wong et al., 2016), but evaluation of the AIS score may require a CT scan and specialist knowledge of injuries, which will present difficulties for the nurses in EDs. Development of an extremity and external injury score variable, which can be easily used by nurses, will be included in future research. As shown in Table 2, the MEWS-A’s ability to predict injury severity is marginally better than that of the MEWS because the majority of the cohort’s injury sites are the head and neck. Additional data on patients presenting with chest and abdominal injuries will also be collected in future research.

The present study also showed the optimal cut-off threshold of the MEWS-A score ≥4 for identifying injury severity in multiple trauma patients with high specificity (0.93) and good sensitivity (0.54). The MEWS-A ≥4 can be used in decision-making protocol for ICU admission, surgery, and immediate medical intervention.

The MEWS-A score can be rapidly calculated even though incorporating an abdomen score increases the complexity of the scoring system. The abdomen score can be directly measured through palpation, avoiding the challenges associated with the requirement of special medical devices and transportation of patients between rooms and floors for examination. Empirically, nurses can accomplish the assessment of the MEWS-A within 5 min of the patient’s arrival at the ED, thus requiring much less time than the 30 min needed to determine the ETS (Raum et al., 2009). Hence, the MEWS-A is convenient and can be used for multiple trauma triage in the ED.

Our study has several limitations. First, it was retrospective and was limited to a relatively small sample of patients from a single center. Owing to the retrospective design, it is possible that the calculated scores may have been different had they been obtained in real time because of the time pressure experienced at EDs and the variability due to scoring by multiple individuals. A prospective study based on a larger cohort from multiple emergency centers is, therefore, necessary to validate the findings. Most multiple injury patients in the cohort presented with head and neck injuries, which may cause bias in the results. In future research, more medical records of multiple trauma patients should be analyzed for further evaluation of injury severity assessment using the MEWS and MEWS-A score.

## Conclusions

The conventional MEWS is suitable for predicting mortality but is a relatively poor predictor of injury severity. However, the MEWS-A score, which incorporates an abdomen score into the conventional MEWS, is superior in identifying injury severity in multiple trauma patients, indicating that incorporating a site-specific injury severity score (e.g., abdomen score) can improve the performance of the MEWS. The MEWS-A is a simple and rapid tool for application and can be used for the timely triage of multiple trauma patients in the ED.

##  Supplemental Information

10.7717/peerj.10242/supp-1 Supplemental Information 1 Study cohort raw data

10.7717/peerj.10242/supp-2 Supplemental Information 2 Code book

## Additional Information and Declarations

### Data Availability

The following information was supplied regarding data availability: Raw data and a code book are available in the Supplementary Files.
